# Endoscopic balloon dilation for peptic gastroduodenal stenosis with gastric outflow obstruction: effectiveness, durability and early predictors of unsatisfactory outcomes

**DOI:** 10.1186/s12876-026-04619-6

**Published:** 2026-01-14

**Authors:** Adam Zeyara, Léonie Scarfone, Martin Jeremiasen, Dan Falkenback, Bodil Andersson, Bobby Tingstedt, Jan Johansson

**Affiliations:** 1 Department of Clinical Sciences, Lund University, Lund, Sweden; 2 Department of Surgery, Skåne University Hospital, Lund, Sweden

**Keywords:** Endoscopic balloon dilation, Peptic, Stenosis, Gastric, Outflow, Obstruction

## Abstract

**Background:**

Peptic gastroduodenal stenosis is a rare but disabling condition causing gastric outlet obstruction. This study aimed to evaluate endoscopic balloon dilation in terms of treatment effectiveness, durability, and identify potential early predictors of unsatisfactory outcomes.

**Methods:**

We conducted a retrospective cohort study of patients who underwent endoscopic balloon dilation for symptomatic peptic gastroduodenal stenosis at Skåne University Hospital, Lund, Sweden between January 1st, 2003, and December 31st, 2023.

**Results:**

A total of 58 patients were included, with a median follow-up of 9.58 years [IQR 4.38–14.15]. Endoscopic balloon dilation was successful in 50 patients (86.2%), of whom 7 (14%) experienced symptomatic recurrence that was managed with repeat dilations. Eight patients were classified as non-responders and subsequently underwent surgical treatment. Kaplan–Meier curves identified three distinct response patterns among the responders: fast responders (0–1 months), intermediate responders (1-8 months), and slow responders (8-35 months), roughly corresponding to 1-2 dilations, 3-5 dilations, and 6 or more dilations, respectively. Undergoing more than five dilations had a significantly longer treatment duration and lower treatment effect compared with five or fewer dilations. Moreover, more than five dilations were associated with a significantly increased risk for recurrence (OR = 4.09; 95% CI 1.02–16.40, *p* = 0.047).

**Conclusion:**

Most patients with peptic stenosis causing gastric outflow obstruction can be treated successfully with endoscopic balloon dilation alone. More than five dilations seem to be associated with a prolonged treatment period, lower treatment effect and increased risk for recurrence. These findings suggest that reconsideration of treatment strategy could be considered in patients who fail to improve after five dilations.

## Introduction

Peptic gastroduodenal stenosis is a rare but disabling condition of the antropyloric region of the stomach and the duodenal bulb, resulting in gastric outlet obstruction. Typical symptoms include early satiety, abdominal pain, vomiting and weight loss [1]. Peptic ulcer disease remains the predominant cause in adults, and *Helicobacter pylori* continues to play a major role in both pathogenesis and prognosis [1, 2]. However, following the recognition of the association between *Helicobacter pylori* infection and peptic ulcer disease, the incidence of this complication has markedly declined, with fewer than 5% of bulbar ulcers and fewer than 1–2% of antropyloric ulcers progressing to stenosis [3].

Surgical intervention historically represented the mainstay of treatment for this condition but has been gradually supplanted by endoscopic balloon dilation since it was first described by Benjamin et al. in 1984 [4]. Thence onwards, multiple studies have reported on the use of endoscopic balloon dilation for this condition, with widely varying results in terms of effectiveness, durability, and complication rates. Current guidelines from the American Society of Gastrointestinal Endoscopy (ASGE) recommend endoscopic balloon dilation as the first-line treatment for all benign causes of gastric outlet obstruction, reserving surgical intervention for salvage cases only [5]. However, much of the literature supporting these recommendations is somewhat dated and limited by either small cohort sizes or relatively short follow-up [3, 6–12]. Moreover, there is a lack of clear guidance in terms of when to change trajectory from endoscopic treatment towards surgical intervention.

The aims of this study were therefore to evaluate endoscopic balloon dilation for peptic gastroduodenal stenosis in terms of effectiveness (therapeutic success), long-term durability (symptomatic recurrence or not), and to identify potential early predictors of unsatisfactory outcomes, including symptomatic recurrence.

## Methods

### Manuscript preparation

The manuscript was prepared using the Strengthening the Reporting of Observational Studies in Epidemiology (STROBE) statement [13].

### Study design

Retrospective observational cohort study.

### Study population

Patients eligible for the study had undergone an endoscopic balloon dilation for symptomatic gastric outlet obstruction due to peptic gastroduodenal stenosis localized in the antropyloric region of the stomach or the duodenal bulb at Skane University Hospital in Lund, Sweden between January 1st, 2003, and December 31st, 2023.

Exclusion criteria were < 18 years, pregnancy, asymptomatic stenosis, non-peptic stenosis (including malignancy, stenosis secondary to previous surgical interventions, or other non-peptic causes such as corrosive injury or Crohn’s disease), and patients still undergoing their first dilation series at the time of data collection. Stenoses were classified as peptic if none of these exclusion criteria were present.

### Data collection

Electronic records of the included patients were searched and predetermined variables extracted into a predesigned spreadsheet using the IBM SPSS Statistics 30.0 (Build 172) software.

### Outcome measures

#### Primary outcome

- Treatment effect of endoscopic balloon dilation.

#### Secondary outcomes

- Long-term results of endoscopic balloon dilation.
- Potential predictors of unsatisfactory outcomes.

### Definitions and special considerations

- *Dilation series* was defined as a series of dilations until stopped for any reason. If more than 12 months elapsed between dilations it was defined as a new dilation series due to recurrence.
- *Responder* was defined as a patient who achieved sufficient treatment effect to discontinue dilation treatment, either on the treating physician’s recommendation or at the patient’s own discretion.
- *Non-responder* was defined as a patient for whom a change of strategy for example to surgery was made due to unsatisfactory treatment effect.
- *Recurrence* was defined as recurrence of typical symptoms at least 12 months after the last dilation and requiring a new dilatation series.
- Complications were graded according to the Clavien-Dindo classification [14].

### Procedural details

All procedures were performed by referral-center-level surgeon-endoscopists with expertise in both endoscopic and surgical management of gastric outflow obstruction. Standard adult Olympus^®^ gastrointestinal endoscopes were consistently used throughout the study period; while specific models varied over time, endoscope outer diameters ranged from approximately 9.6–10 mm in the early 2000s to 9.2 mm in more recent years. Endoscopic balloon dilations were performed using through-the-scope balloon dilators without fluoroscopic guidance. Commercially available balloon systems from Boston Scientific^®^ were consistently used. No major changes in this practice were introduced during the study period.

### Statistics

#### Basic descriptive and comparative analyses

Categorical and continuous variables were treated accordingly. Categorical data were compared using the χ² or Fisher’s exact test, as appropriate. Continuous variables were expressed as mean or median depending on distribution and compared using parametric or non-parametric tests as appropriate. Statistical significance was set at *p* < 0.05.

#### Kaplan-Meier and regression analyses

Responders were analyzed using Kaplan–Meier curves, plotting cumulative treatment effect over treatment time. The Log-rank test was used to determine whether time-to-event differences were statistically significant or not.

Regression analyses were planned a priori to explore associations between predefined patient-, disease- and procedure-related variables and treatment outcomes. Logistic regression analysis was used to compare responders and non-responders as the primary binary outcome. When applicable, results were expressed as odds ratios (OR) with 95% confidence intervals (CI), significance set at *p* < 0.05. Cox proportional hazards regression was used to explore potential predictors of time to treatment success. When applicable, results were presented as hazard ratios (HR) and 95% CI, significance set at *p* < 0.05. The variables that were entered into these models were selected a priori based on clinical relevance and included demographic characteristics, comorbidities, stenosis characteristics, *Helicobacter pylori* status, medication use, and technical aspects of the dilation procedure.

Following Kaplan-Meier analyses, additional exploratory analyses were performed to further examine the relationship of treatment intensity (number of dilations) with clinical outcome. Associations with outcome measures were assessed using Fisher’s exact test, and unadjusted logistic regression was used to estimate effect sizes.

#### Missing data

Missing data in crucial variables resulted in complete-case exclusion for the affected observation.

#### Software

All statistical analyses were performed using the IBM SPSS Statistics 30.0 (Build 172) software for Mac.

## Results

**Fig. 1:**
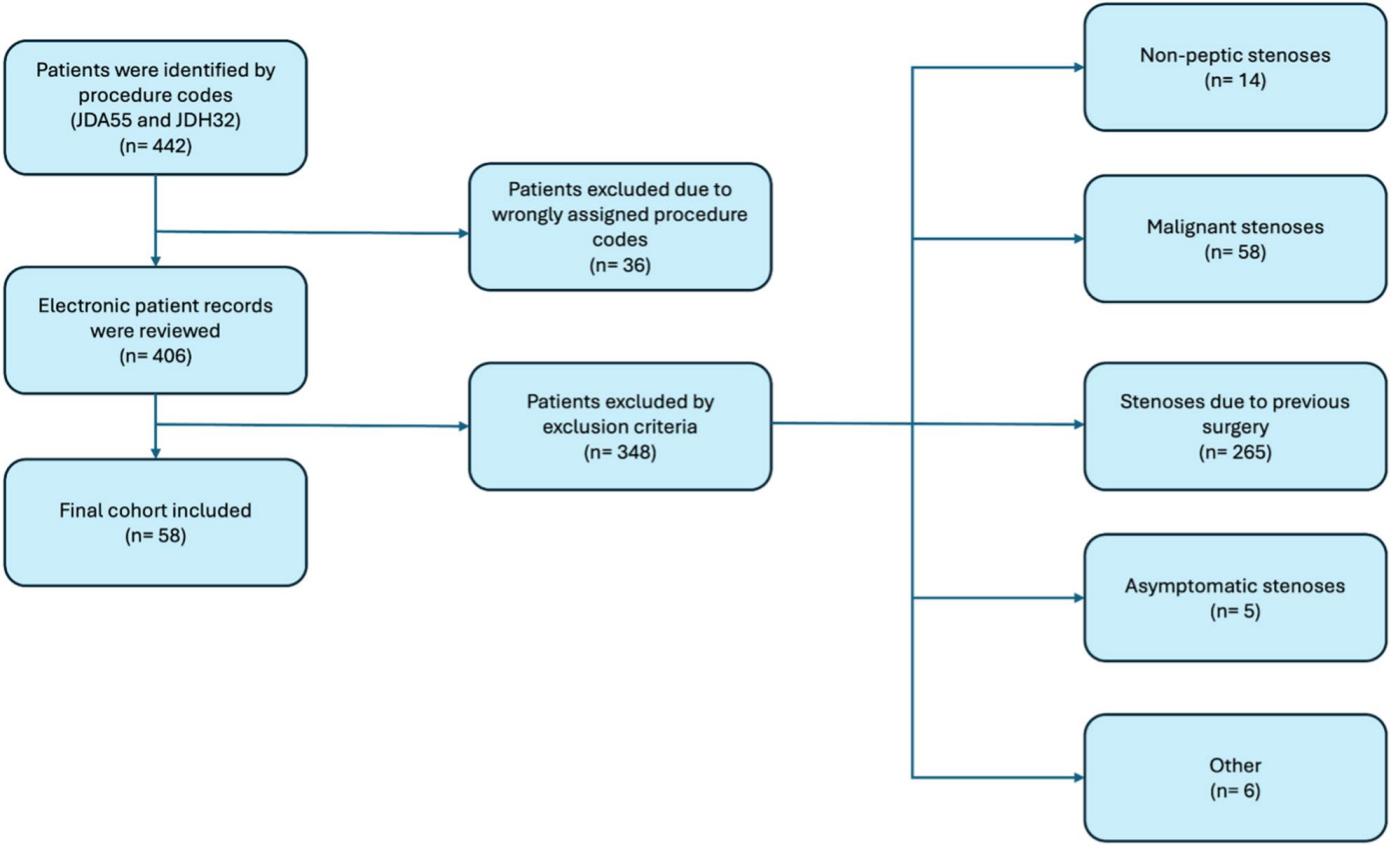
Patient inclusion process: A total of 442 electronic patient records were reviewed. Of these, 36 were excluded due to incorrect procedure coding and 348 were excluded based on previously established criteria. Ultimately, 58 patients were eligible for inclusion

A total of 442 electronic patient records were reviewed. Of these, 36 were excluded due to incorrect procedure coding, and 348 were excluded based on previously established criteria. Ultimately, 58 patients were eligible for inclusion. The patient inclusion process is illustrated in Fig. 1.

Among the 58 included patients, 50 (86.2%) showed a satisfactory clinical response after the first dilation series. Of these, seven patients (14%) experienced symptomatic recurrence, which was successfully managed with additional dilation series. None of these patients required surgery. Eight patients (13.6%) were classified as non-responders and subsequently underwent surgical intervention—either partial distal gastrectomy or simple gastrojejunostomy. Overall complications and Clavien-Dindo *≥* 3 complications occurred in 16 (28%) and 7 (12%) patients, respectively. The median follow-up time was 9.58 years [IQR 4.38–14.15]. A summary of these outcome data is presented in Table 1.

**Table 1:** General outcome data

Responders (effectiveness), *n* (% of total 58)	50 (86.2)
Non-responders, *n* (% of total 58)	8 (13.8)
Symptomatic recurrence in responder group, *n* (% of subtota 50)	7 (14)
Complications, *n* (% out of total 58)	
All	16 (28)
Bleeding	10
Perforation	4
Clavien-Dindo >3	7 (12)
Median follow-up time, years [IQR]	9.58 [4.38 – 14.15]

Patients were categorized as responders (*n* = 50) or non-responders (*n* = 8). Significance testing revealed no statistically significant differences between the groups in terms of both patient characteristics and procedural variables (Tables 2 and 3). Regression analyses comparing responders and non-responders did not identify any statistically significant associations and effect estimates are therefore not reported.

**Table 2:** Descriptive patient data, responders versus non-responders: the table depicts descriptive data after dividing the patients into responders (*n* = 50) and non-responders (*n* = 8). Significance tests did not show any significant differences between the groups in the included variables

	Responders (*n* = 50)	Non-responders (*n* = 8)	*p*-value
Gender, *n* (%)			0.071
Male	23 (46)	3 (37.5)	
Female	27 (54)	5 (62.5)	
Mean age, years (± SD)	68.4 (± 12.8)	61.6 (± 16.2)	0.976
Mean BMI, kg/m^2^ (± SD)	23.6 (± 4)	23 (± 4)	0.599
ASA *≥* 3, *n* (%)	21 (42)	4 (50)	0.478
Smoking, *n* (%)			0.091
Yes	14 (28)	1 (12.5)	
No	32 (64)	6 (75)	
Unknown	2 (8)	1 (12.5)	
Alcohol, *n* (%)			0.132
Yes	3 (6)	0 (0)	
No	28 (56)	6 (75)	
Unknown	19 (38)	2 (25)	
Chronic NSAID, Aspirin or steroid use before diagnosis, *n* (%)	34 (68)	6 (75)	0.090
Completed *Helicobacter pylori* eradication after diagnosis, *n* (%)			0.067
Yes	35 (70)	4 (50)	
Confirmed	27	4	
Unconfirmed	8	0	
Exact stenosis locale, *n* (%)			0.071
Antropyloric stomach	35 (70)	4 (50)	
Duodenal bulb	15 (30)	4 (50)	

**Table 3:** Procedure-specific data responders versus non-responders: the table depicts descriptive data after dividing the patients into responders (*n* = 50) and non-responders (*n* = 8). Significance tests did not show any significant differences between the groups in the included variables

	Responders (*n* = 50)	Non-responders (*n* = 8)	*p*-value
Mean total number of dilations in first series, *n* (± SD)	3.9 (± 3.1)	2.9 (± 2.6)	0.709
Mean dilation intervals in first series, days (± SD)	55 (± 56.1)	44.7 (± 43.7)	0.678
Mean total time of first series, months (± SD)	3.3 (± 4.4)	3.0 (± 3.6)	0.879
Mean maximum balloon dimension in first series, mm (± SD)	14.7 (± 3.7)	13.3 (± 2.5)	0.309
Stenosis passable with standard adult endoscope on diagnosis, *n* (%)	13 (26)	0 (0)	0.630
Stenosis passable with standard adult endoscope after completion or abortion of first dilation series, *n* (%)	36 (72)	4 (50)	0.063

**Fig. 2:**
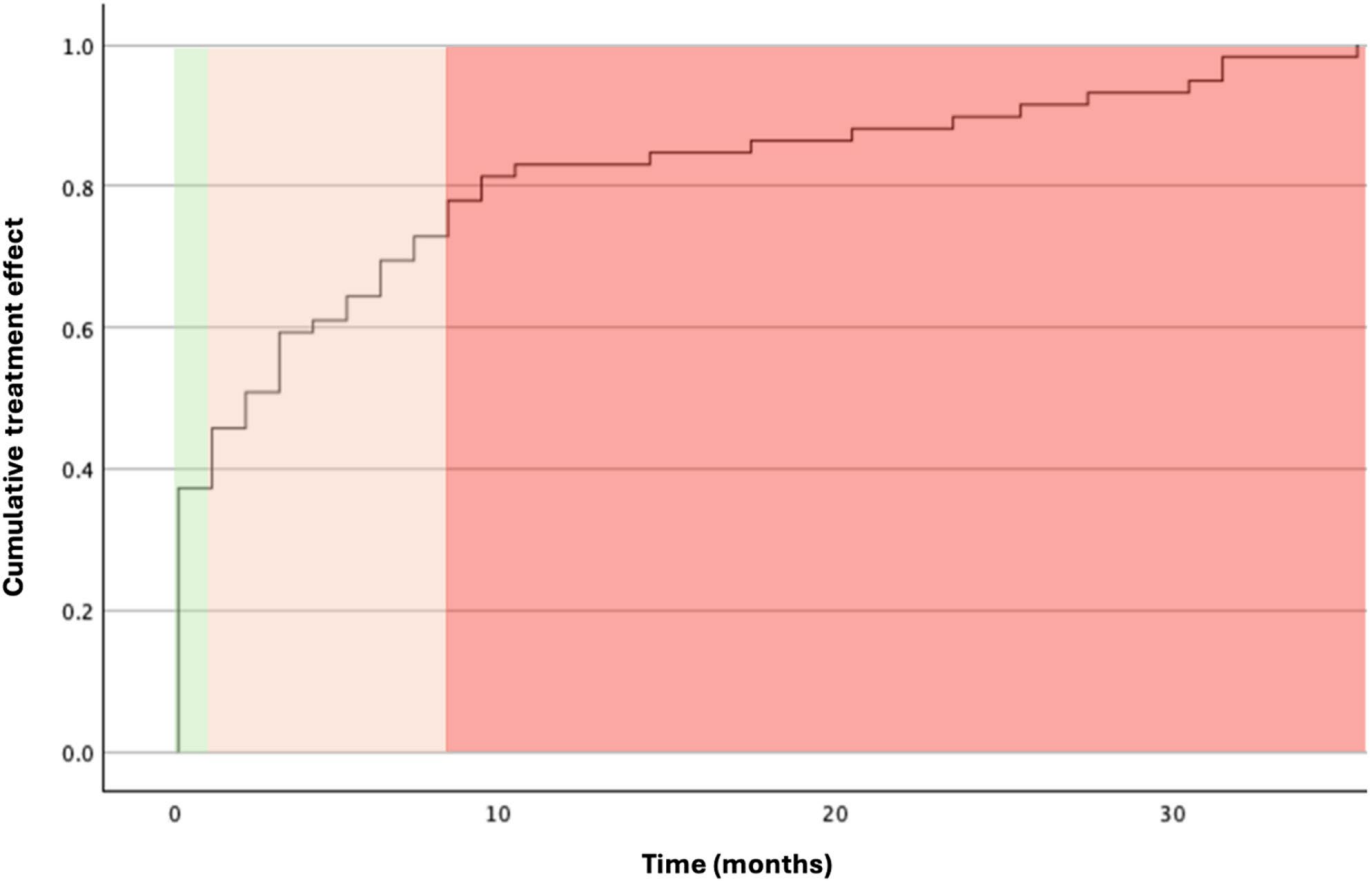
Cumulative Kaplan–Meier curve showing time to treatment success. Three response groups were identified: early (0–1 month, ~ 40%), intermediate (1–8 months, ~ 40%), and late responders (8–35 months, ~ 20%). The analysis was restricted to responders only

A cumulative Kaplan–Meier curve was plotted with time to treatment effect (months) on the X-axis and the cumulative treatment effect on the Y-axis (Fig. 2). The graph revealed three distinct response groups: (1) approximately 40% of patients responded within 0–1 month, (2) another 40% within 1–8 months, and (3) the remaining 20% within 8–35 months. The analysis was restricted to responders only.

**Fig. 3:**
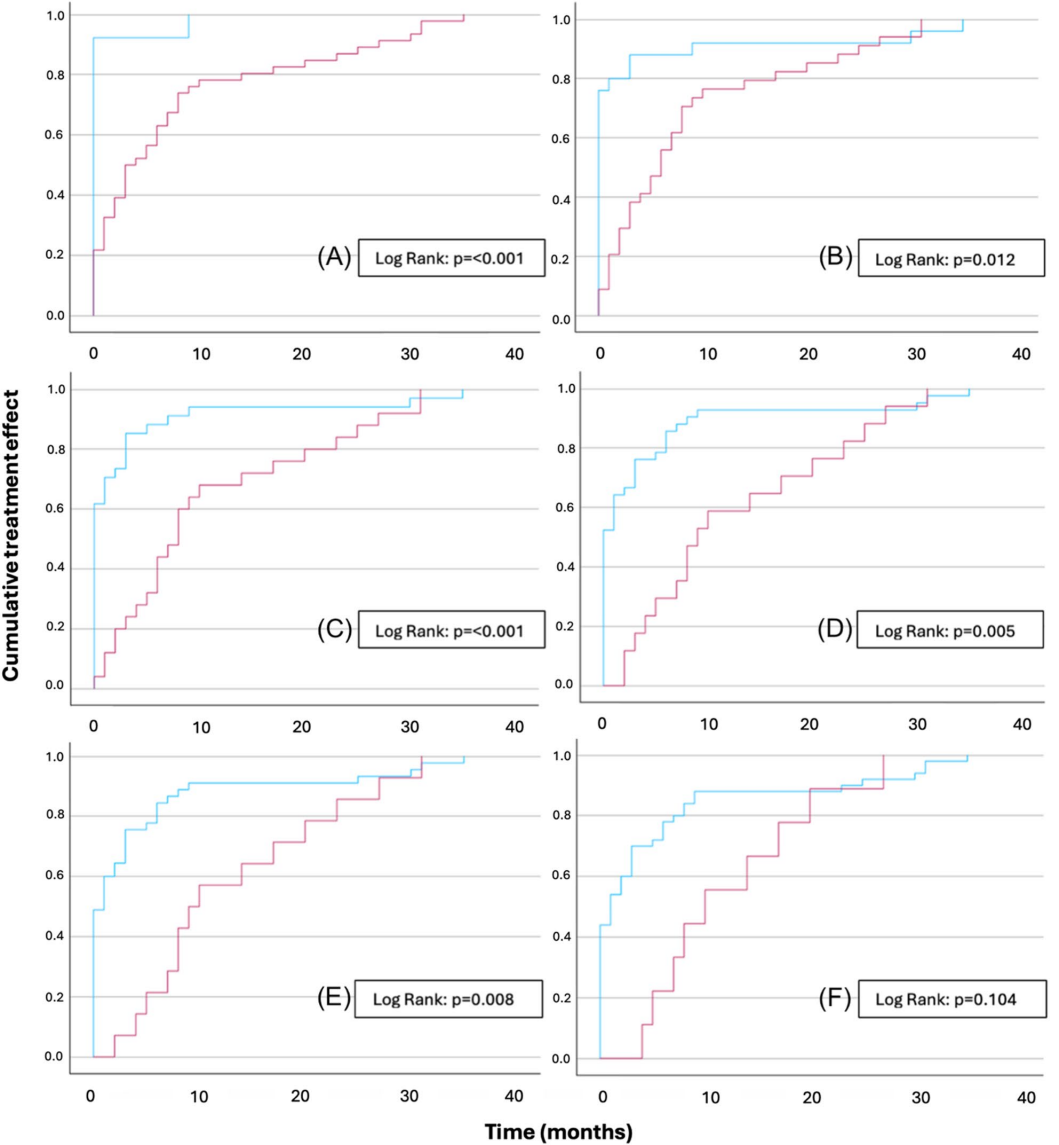
Dichotomized Kaplan–Meier curves comparing cumulative treatment effect for increasing dilation thresholds: (**A**) 1 dilation (blue) vs. ≥ 2 dilations (red), (**B**) 1–2 (blue) vs. ≥ 3 (red), (**C**) 1–3 (blue) vs. ≥ 4 (red), (**D**) 1–4 (blue) vs. ≥ 5 (red), (**E**) 1–5 (blue) vs. ≥ 6 (red), and (**F**) 1–6 (blue) vs. ≥ 7 (red) dilations. Log-rank tests showed significantly longer treatment time with each added dilation up to five, beyond which efficacy declined, suggesting a clinical distinction between ≤ 5 and > 5 dilations. The analysis was restricted to responders only

To further analyze treatment dynamics, dichotomized Kaplan–Meier curves were generated comparing different cutoff points for the number of dilations: 1 vs. ≥ 2 (Fig. 3A), 1–2 vs. ≥ 3 (Fig. 3B), 1–3 vs. ≥ 4 (Fig. 3C), 1–4 vs. ≥ 5 (Fig. 3D), 1–5 vs. ≥ 6 (Fig. 3E), and 1–6 vs. ≥ 7 (Fig. 3F). Log-rank tests showed a significant increase in treatment time for each additional dilation up to five, indicating that beyond five dilations, a longer treatment duration and diminished treatment effect could be expected. Two patients in the two-dilation group had prolonged treatment durations due to the predefined requirement of > 12 months before recurrence was recorded, resulting in artificially extended time-to-event estimates. The analysis was restricted to responders only.

Cox regression analyses incorporating multiple patient-, disease- and procedure-related variables – including demographics, comorbidities, stenosis characteristics, *Helicobacter pylori* status, medication use, and technical aspects – did not identify any additional significant predictors of treatment outcome and effect estimates are therefore not reported.

The Kaplan-Meier analyses did however suggest potential underlying differences between the patients who required five or less dilations and those who required more than five, prompting further investigation. Fisher’s exact test demonstrated a significant association between requiring more than five dilations and recurrence (*p* < 0.001). In unadjusted logistic regression, patients undergoing more than five dilations had higher odds of recurrence (OR 4.09; 95% CI: 1.02–16.40, *p* = 0.047). Stepwise backward elimination including all clinically relevant covariates did not materially alter the direction or statistical significance of this association but was ultimately limited by the low number of recurrent events, precluding reliable multivariate inference and estimation of independent predictors.

**Fig. 4:**
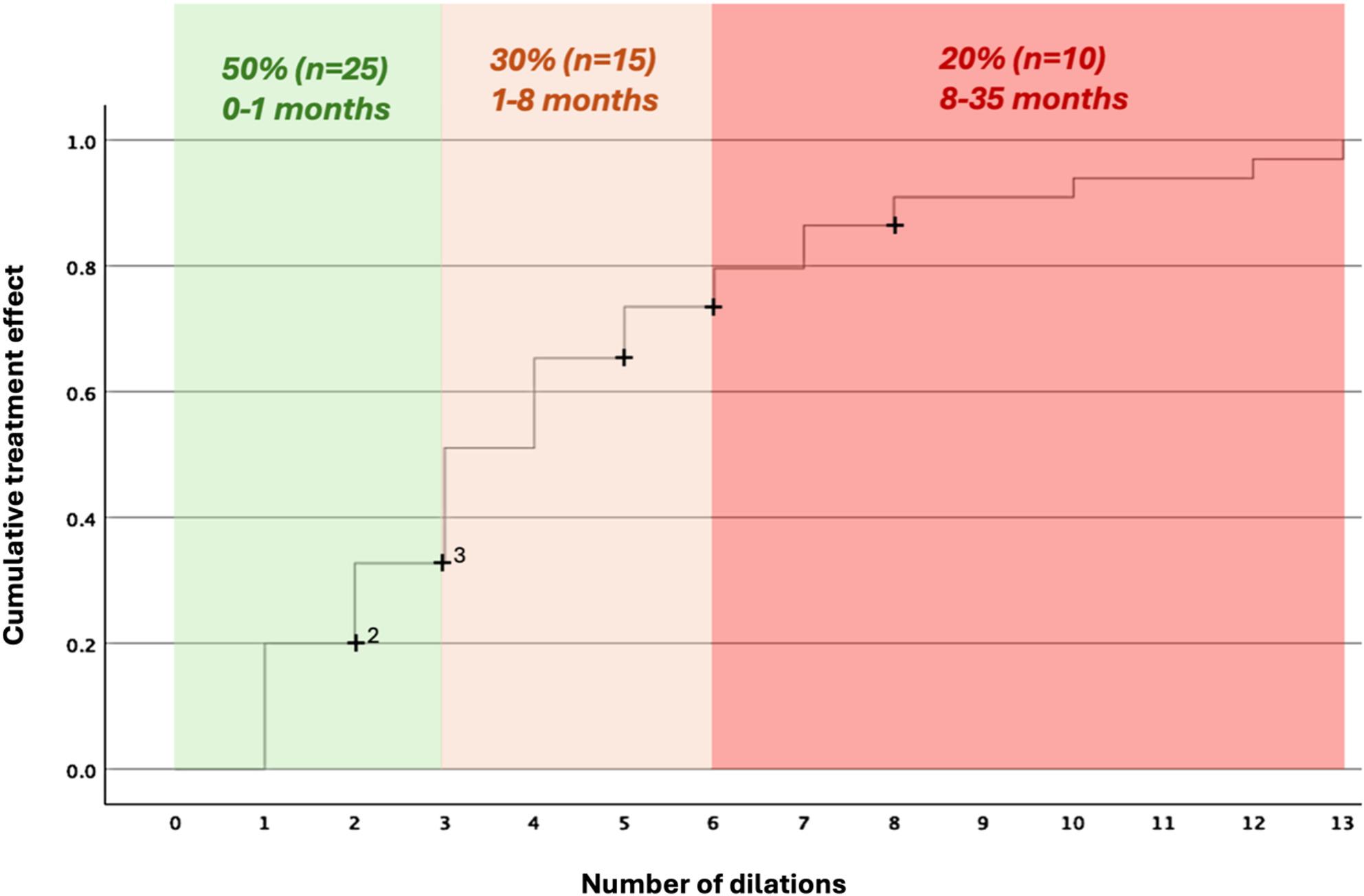
Kaplan–Meier curve illustrating treatment success by number of dilations (used as a surrogate for time). Curve steepness decreased markedly after five dilations, consistent with previous analyses. Three response zones were identified: fast responders (1–2 dilations, 0–1 month, 50%), intermediate responders (3–5 dilations, 1–8 months, 30%), and slow responders (≥6 dilations, 8–35 months, 20%). The analysis was restricted to responders; non-responders were censored at the time of surgical intervention

Finally, to provide a more clinically intuitive visualization of the findings, an additional Kaplan–Meier curve was plotted (Fig. 4). It displays the cumulative treatment effect on the Y-axis and, as a surrogate for time, the number of dilations until treatment effect was achieved on the X-axis. Visual inspection revealed a marked flattening of the curve after five dilations, consistent with the previous analyses (Fig. 3). Building on the three response groups identified in Fig. 2, color zones were added to represent corresponding response categories. The exact proportions were slightly adjusted to match whole dilation counts. The final visualization delineated three clinical response zones:

1. Fast responders: 1–2 dilations (0–1 months), ~50% of patients.
2. Intermediate responders: 3–5 dilations (1–8 months), ~30% of patients.
3. Slow responders: ≥6 dilations (8–35 months), ~20% of patients.

The analysis was restricted to responders, with non-responders censored at the time of surgical intervention. 

## Discussion

In this series, endoscopic balloon dilation achieved an effectiveness rate of 86.2% after the first dilation series, with long-term durability (no recurrences) of 74%. The overall complication rate was 28%, of which 12% were classified as Clavien-Dindo grade *≥* 3. The factor most strongly associated with recurrence was the need for more than five dilations.

Comparisons with previous studies are generally challenging due to the small size of most published series (only two included more than 25 patients) and heterogeneity in definitions and outcome measures. Nevertheless, reported immediate effectiveness rates in the literature range up to > 90%, recurrence rates of up to 84%, and complication rates are generally < 10%; however, complications are variably defined, and many studies primarily report major adverse events, which may partly explain the higher overall complication rate observed in the present cohort [3, 6–12].

A key finding of this study was the identification of three distinct response patterns: fast responders (0–1 months), intermediate responders (1–8 months), and slow responders (8–35 months), roughly corresponding to 1–2 dilations, 3–5 dilations, and five and more dilations, respectively. Patients undergoing more than five dilations experienced significantly longer treatment durations and lower therapeutic effectiveness compared with those treated with five or fewer dilations. Furthermore, requiring more than five dilations was associated with significantly increased odds of clinical recurrence, regardless of other seemingly relevant clinical variables.

In contrast to several previous publications, non-responders in the present study had a relatively short treatment period and few dilations before proceeding to surgical intervention [3, 6–12]. One plausible explanation is that these patients exhibited very early indicators of treatment failure or more severe symptoms, prompting earlier escalation to surgery. Supporting this interpretation, none of the stenoses in the non-responder group were passable with a standard endoscope at the time of diagnosis. An alternative explanation may relate to provider-treatment bias, whereby gastroenterologists may be more inclined to persist with endoscopic management, whereas surgeon-endoscopists may favor earlier surgical intervention in the presence of severe or progressive symptoms [15–19]. In the present study, all procedures were performed by referral center level upper gastrointestinal surgeons, all skilled in both endoscopic and surgical treatment of this condition, a unique characteristic among published series that may have mitigated this potential bias.

To the best of our knowledge, this represents the largest available series in English-language literature evaluating endoscopic balloon dilation for peptic gastroduodenal stenosis with gastric outlet obstruction, with an unprecedented duration of follow-up. Nevertheless, it remains underpowered in several aspects and suffers from typical weaknesses inherent to all retrospective studies. For example, some of the comparative groups are very small, limiting statistical precision due to type I/II errors, lack of representativeness, violation of statistical assumptions, overfitting in modeling etc. Additionally, retrospective assessment of patient-reported outcomes required subjective interpretation of medical records; however, all evaluations were performed by the same consultant gastrointestinal surgeon to ensure internal consistency. As with other studies in this field, the findings may also contribute to cumulative publication bias.

We conclude that endoscopic balloon dilation is an effective treatment for peptic gastroduodenal stenosis, resulting in symptom resolution and sustained remission in most patients. The need for more than five dilations was strongly associated with significantly prolonged treatment duration, reduced effectiveness, and increased risk of recurrence. These findings suggest that reassessment of treatment strategy may be warranted in patients failing to achieve symptomatic improvement after five dilations.

## Data Availability

All data are available upon reasonable request.

## Abbreviations

SD: standard deviation
IQR: interquartile range
CI: confidence interval
OR: odds ratio
HR: hazard ratio
BMI: body mass index
STROBE: Strengthening the Reporting of Observational Studies in Epidemiology
ASA: American Society of Anesthesiology
NSAID: non-steroidal anti-inflammatory drug
ASGE: American Society of Gastrointestinal Endoscopy
